# The Association of Malnutrition, illness duration, and pre-morbid weight status with anxiety and depression symptoms in adolescents and young adults with restrictive eating disorders: a cross-sectional study

**DOI:** 10.1186/s40337-021-00415-7

**Published:** 2021-05-17

**Authors:** Jessica A. Lin, Grace Jhe, Julia A. Vitagliano, Carly E. Milliren, Rebecca Spigel, Elizabeth R. Woods, Sara F. Forman, Tracy K. Richmond

**Affiliations:** 1 Division of Adolescent/Young Adult Medicine, Boston Children’s Hospital, 300 Longwood Ave, Boston, MA 02115 USA; 2 Harvard Medical School, 25 Shattuck Street, Boston, MA 02115 USA; 3 Institutional Centers for Clinical and Translational Research, Boston Children’s Hospital, 300 Longwood Ave, Boston, MA 02115 USA

**Keywords:** Eating disorders, Anorexia nervosa, Avoidant/restrictive food intake disorder, Malnutrition, Anxiety, Depression, Adolescents, Young adults

## Abstract

**Background:**

Restrictive eating disorders (EDs) are often comorbid with anxiety and depression symptoms, placing patients at risk for more severe disease, worse treatment outcomes, and higher rates of mortality. To identify risks for developing such co-morbidities, we assessed the association of malnutrition, ED illness duration, and pre-morbid weight status with symptoms of anxiety and depression in adolescents/young adults (AYAs) with EDs.

**Methods:**

145 participants with restrictive EDs (anorexia nervosa [AN], other specified feeding and eating disorders [OSFED], avoidant restrictive food intake disorder [ARFID]) were included from the RECOVERY study, a longitudinal web-based registry of AYAs with EDs. We measured malnutrition as percent of expected body mass index (%eBMI), based on participants’ pre-morbid growth trajectory. Outcomes were anxiety and depression scores from the Generalized Anxiety Disorder 7-item (GAD-7) and Center for Epidemiologic Studies Depression (CES-D) scales. We used multiple linear regression to examine the association of malnutrition, ED duration, and pre-morbid weight status with symptoms of anxiety and depression.

**Results:**

Mean (SD) age was 16.4(3.0) years; 87% were female; 89% white; 85% had AN, 6% OSFED, 10% ARFID. Of these, 2/3 had ED symptoms ≥1 year, 1/3 had previous higher level of ED care (HLOC), and half were taking psychiatric medications. Mean %eBMI was 90% (range 57–112%). Mean GAD-7 was 9.4(5.9) and CES-D was 24(13.8), indicating most participants had clinically significant anxiety and/or depression. Degree of malnutrition was not significantly associated with anxiety or depression adjusting for age, sex, sexual orientation, ED diagnosis, and use of psychiatric medication. Those with longer duration of ED symptoms had higher depression scores after adjusting for malnutrition, HLOC, length of ED symptoms, and time in our care (*p =* 0.038). Patients with pre-morbid BMIs ≥75th percentile had lower depression scores than those with pre-morbid BMIs <75th percentile (*p* = 0.014).

**Conclusions:**

We find high degree of clinically relevant anxiety and depression symptoms in a population of AYAs with EDs. Our findings suggest that factors beyond malnutrition play a role in the co-morbid mood and anxiety disorders in this population. Overall, rapid ED diagnosis and comprehensive treatment for patients with EDs across the weight spectrum—and especially those with psychiatric co-morbidities—will likely aid in recovery.

## Plain english summary

People with eating disorders (EDs) often also struggle with anxiety and depression. Unfortunately, this can mean their disease is more severe, harder to treat, and more dangerous and potentially fatal. ED treatment often primarily focuses on nutrition and weight gain, and sometimes loses focus on the person’s mood and anxiety. Additionally, patients can present at any weight, but not enough is known about how baseline weight before weight loss might affect mood. In this study, we focus on adolescents/young adults with restrictive EDs to understand what places them at risk for depression and anxiety. We find that the majority of our participants have clinically significant anxiety and depression. However, degree of malnutrition was not related to level of anxiety and depression. Struggling with an ED for longer time was associated with increased depression and possibly anxiety as well. We also find that those who had relatively high baseline weights prior to weight loss had *lower* depression and anxiety scores than those who had lower baseline weights. Early identification and treatment of individuals with EDs is important, and there are likely other factors beyond malnutrition that we need to understand in order to identify those at risk for depression and anxiety.

## Introduction

Eating disorders (EDs) are psychiatric conditions associated with significant risk of medical complications and high mortality rates [1]. The medical complications of EDs can affect all organ systems and are largely driven by malnutrition [2, 3]. These complications—such as arrythmias [4, 5], heart failure [4, 5], and liver failure [6, 7]—can be fatal [4, 6], but can also be reversed with weight restoration [8]. Thus, nutritional rehabilitation often becomes the primary focus of ED treatment [7, 9, 10]. Individuals with EDs are also at high risk of co-morbid psychiatric disorders, such as affective disorders, anxiety disorders (e.g. generalized anxiety disorder, separation anxiety, social anxiety disorder), and substance use [11–13]. In fact, the increased mortality rates in individuals with EDs is largely due to the co-morbid mental health conditions leading to high rates of suicide attempts and death by suicide [14]. The risk of death by suicide is more than five times higher in individuals with EDs compared to the general population, and is highest in those with comorbid psychiatric illnesses, such as anxiety disorders and major depressive disorder [14]. The presence of mental health comorbidities in individuals with EDs has been found to be associated with more severe ED symptoms [15], worse treatment outcomes [16], and increased mortality [17], suggesting that these psychiatric illnesses pose an immense risk if left untreated. Yet for many, the emphasis of ED-related treatment remains on weight restoration first, leaving co-morbid mental health conditions undertreated [18].

The emphasis on weight restoration is largely based on the belief that malnutrition drives much of the commonly seen mood disturbances and anxiety symptoms in individuals with EDs [19] and that weight restoration will improve mood and anxiety symptoms [19–23]. Good evidence does exist for the importance of increasing nutritional intake and weight restoration for physical recovery and improvement in ED thoughts and behaviors [7–10], but evidence is inconsistent on the effect of malnutrition and weight restoration on co-morbid mental health conditions [19–25]. This limited understanding of the true impact of malnutrition on anxiety and depressive symptoms among individuals with restrictive EDs makes it difficult to determine whether a primary focus on weight restoration indeed provides the best treatment. Additionally, there is limited understanding of what other factors increase the risk of developing co-morbid depression and anxiety disorders that in turn may worsen the course of EDs. The evidence for the benefits of improved nutrition is bolstered by the evidence supporting Family Based Treatment (FBT), a treatment modality which focuses primarily on weight restoration as the route for recovery, especially for adolescents with restrictive EDs [3]. However, despite FBT having some of the best-documented efficacy of any treatment modality and viewed as the gold standard for youth with restrictive EDs, the full remission rate in studies of FBT is still less than 50% [26]. Thus, there are likely other factors that contribute to the lack of recovery in more than half of those seeking treatment. One potential barrier is the under-treatment of co-morbid mood disturbance or anxiety symptoms. Therefore, it is crucial that we identify factors that place individuals at risk for developing psychiatric co-morbidities as well as ED treatment strategies that effectively address mental health co-morbidities in order to improve treatment outcomes.

Currently, the few studies addressing the relationship between malnutrition and anxiety and depression symptoms focus only on individuals with AN; however, malnutrition and *relative* malnutrition (i.e., being well below one’s natural growth pattern) are common in other EDs [1, 27, 28]. Individuals with AN suffer from restrictive eating, fear of weight gain, and distorted body image leading to severe malnutrition, and often have low body mass indices (BMIs) [1]. Those with other restrictive EDs, such as Other Specified Feeding and Eating Disorders (OSFED) or atypical anorexia nervosa (AAN), also experience psychological symptoms, disordered eating behaviors, and/or medical complications related to malnutrition. Their relative malnutrition is easily missed as patients with AAN by definition have a BMI in a Centers for Disease Control and Prevention (CDC)-defined ‘healthy’ weight range after unsafe weight loss [1, 29]. Patients with AAN have the same rates of medical complications and anxiety and depression symptoms as those with AN, but are more likely to experience severe psychosocial distress related to eating and body image [28, 30]. Because these patients are often missed early in their disease course, they tend to present to care later and with more significant weight loss [31, 32]. Patients with Avoidant/restrictive Food Intake Disorder (ARFID), a restrictive ED recently recognized in the DSM-5 [1], can also have profound malnutrition. These individuals struggle with inadequate intake due to disinterest, fear of an adverse event when eating, or aversion to certain sensory characteristics of food [1]. As with other restrictive EDs, ARFID is associated with severe malnutrition and significant psychological distress [27, 33]. Therefore, it is crucial that we clarify how malnutrition is associated with anxiety and depression symptoms across the spectrum of restrictive EDs (i.e., AN, OSFED, and ARFID) and across the weight spectrum.

In addition to the importance of nutritional rehabilitation, early treatment leading to early weight restoration is associated with improved morbidity and mortality [9, 34]. However, little data is available regarding the impact of rapid nutritional rehabilitation on changes in anxiety and depression symptoms, which may be a potential mechanism through which to improve ED outcomes.

In order to address the identified gaps in the literature, we aim to examine the association of the following ED-related characteristics with anxiety and depressive symptoms in adolescents and young adults with restrictive EDs: 1. degree of malnutrition; 2. duration of ED symptoms; and 3. pre-morbid BMI status (i.e., elevated pre-morbid BMI v. not). We hypothesize that more severe malnutrition and longer duration of ED symptoms will be associated with higher anxiety and depression scores. We further hypothesize that individuals with higher pre-morbid weight status will have more severe anxiety and depression symptoms compared to those with pre-morbid weight in a ‘healthy’ range.

## Methods

### Recruitment

Participants were recruited to enroll in the Registry of Eating Disorders and their Co-morbidities OVER time in Youth (RECOVERY) during a visit to the Boston Children’s Hospital’s (BCH) Outpatient Eating Disorder Program between 2017 and 2020. RECOVERY is a web-based, prospective, longitudinal study of youth and young adults with EDs. Participants between the ages of 10 and 27, with a diagnosed ED, seeking care in the BCH ED Program were eligible. For the present study, we included only those participants diagnosed with restrictive ED diagnoses (AN, OSFED/AAN, and ARFID). One patient completed their baseline survey during the COVID-19 pandemic, and was excluded due to the potential confounding effect of the COVID-19 pandemic on mental health outcomes. Informed consent/assent was obtained by a research assistant in person or by phone. Patients ≥18 years old provided consent. Patients < 18 years old provided assent and a parent/guardian provided consent. This study was approved by the BCH Institutional Review Board.

### Data collection

RECOVERY participants were e-mailed secure links to online surveys via RedCap. Surveys were sent at baseline and every 3 months in the first year after recruitment, and every 6 months thereafter for up to 3 years of participation. Participants were given remuneration in the form of $20 gift cards after each survey completed and could receive up to $180 throughout the course of the study depending on the number of surveys completed. Only the baseline survey data were used for this study. Chart review of the electronic medical records were used to determine the ED diagnosis given by a medical provider, to obtain the most recent BMI corresponding to each survey, and to determine patients’ pre-morbid BMI and growth trajectory. Chart review data were confirmed by two reviewers.

### Measures

#### Degree of malnutrition

Measured by the percentage of expected BMI (eBMI), calculated by taking the ratio of most recent BMI to the BMI for age at the growth curve expected (e.g., if patient grew at 75th percentile in the past, their eBMI would be the 75th percentile). If patients fluctuated between two curves (e.g., 50-75th percentile) prior to the ED onset, an average would be used as their eBMI (e.g., 62.5 percentile). %eBMI was used to capture the spectrum of malnutrition occurring at all presenting BMIs, and also to capture malnutrition in those who had stalled weight gain rather than weight loss. Severity of malnutrition was then categorized into three groups based on the sample’s 25th and 75th percentile of %eBMIs (84.5 and 96%eBMI). This was then rounded to clinically reasonable cutoffs: moderate/severe malnutrition = < 85%eBMI, mild/moderate = 85–94.9%, and weight restored = ≥95%.

#### Length of ED symptoms

Obtained by self-report in the baseline survey. Participants were given five options that assessed for length of their ED symptoms, ranging from < 1 month to ≥3 years. For analysis, answers were collapsed into three clinically meaningful categories: ≤6 months, 7–12 months, and > 1 year.

#### Pre-morbid BMI status

Determined by the patients’ BMI percentile along which they had grown prior to the restrictive ED behaviors and weight loss. Participants were divided into those who had a pre-morbid BMI percentile ≥75th percentile or < 75th percentile.

#### Anxiety

Measured by the Generalized Anxiety Disorder 7-item (GAD-7) scale, a well-validated tool used to screen for anxiety in adolescents and young adults [35, 36]. The total score for GAD-7 (range 0–21) was examined as a continuous variable (with higher scores indicating more severe anxiety). The standard cut off of 10 indicates clinically meaningful anxiety [35, 36].

#### Depression symptoms

Measured by the Center for Epidemiologic Studies Depression (CES-D) scale, a 20-item, well-validated tool used to screen for and monitor symptoms of depression in adolescents and young adults [37, 38]. The total score for the CES-D (range 0–60) was examined as a continuous variable (with higher scores indicating more severe depressive symptoms). A cut off of 16 indicates clinically meaningful depression [37, 38].

##### Demographics

Age, race/ethnicity, sex assigned at birth, and sexual orientation were self-reported in the baseline survey. We included two other self-reported measures from the baseline survey: use of a psychiatric medication and previous higher-level ED treatment (e.g. partial hospitalization or residential level of care). Lastly, the length of time under the care of our ED program was determined by the days between their first ED clinic appointment and when they submitted the baseline survey.

### Analysis

We described our sample using frequencies for categorical variables and means (standard deviation) for continuous variables. We addressed our aims first with bivariate analyses using ANOVA, and then with the following linear regression models to adjust for potential confounders. Our two dependent variables (anxiety [GAD-7] and depression symptoms [CES-D]) were examined separately as continuous variables with normal distributions. All analyses were performed using IBM SPSS v24.

1. *The association of malnutrition with anxiety and depression symptoms.*
  This model adjusted for age, sex assigned at birth, sexual orientation, ED diagnosis, and use of psychiatric medication.
2. *The association of duration of ED illness and anxiety and depression symptoms.*
  This model adjusted for prior ED treatment and severity of illness by including degree of malnutrition, past higher level of ED care (HLOC), and time in our care.
3. *The association of pre-morbid BMI status and anxiety and depression symptoms.*
  Using a stepwise method, this model controlled for age, malnutrition, time in our care, and ED diagnosis.

## Results

### Sample characteristics (see Table 1)

Our sample was made up of the 145 participants in the RECOVERY registry who had a diagnosis of a restrictive ED. Mean (SD) age was 16.4 (3.0) years. The majority of our sample were female (*n* = 126, 87%) and white (*n* = 129, 89%). Eighty-five percent of our participants had a diagnosis of AN (*n* = 123), 6% (*n* = 8) OSFED, and 10% (*n* = 14) ARFID. Nearly two-thirds (*n* = 93, 64%) of patients had ED symptoms for over one year, one-third (*n* = 49, 34%) had previous experience with HLOC, and nearly half (*n* = 67, 46%) were taking a psychiatric medication. Mean percentage of eBMI was 89.7% with a range of 57–112% eBMI. Twenty-six percent of participants had moderate/severe malnutrition, 43% had mild/moderate malnutrition, and 30% were weight restored at the time of baseline survey. Of all 145 eligible participants, 142 participants completed the GAD-7 and 140 completed the CES-D, and were ultimately included in the multivariate analyses. Mean GAD-7 score was 9.4 (5.9) and CES-D score was 24 (13.8), with nearly half of those who completed these measures (*n* = 69, 49%) having clinically significant anxiety defined by a GAD-7 score of 10 or greater and 70% (*n* = 99) having a clinically significant CES-D score ≥ 16.

**Table 1:** Sample demographics and eating disorder characteristics (*N* = 145 unless otherwise stated)

	Mean (SD) or n (%)
Age	16.4 (3.0)
Sex assigned at birth	
Female	126 (87%)
Race	
White	129 (89%)
Ethnicity	
Hispanic/Latinx	11 (8%)
Sexual Orientation (*n* = 143)	
Heterosexual	91 (63%)
ED Diagnosis	
Anorexia Nervosa	123 (85%)
OSFED	8 (6%)
ARFID	14 (10%)
% of Expected BMI	89.7 (9.7)
Duration of ED Symptoms (*n* = 141)	
≤ 6 months	27 (19%)
7–12 months	21 (15%)
> 1 year	93 (65%)
Previous HLOC	
Yes	49 (34%)
Taking psychiatric medication (*n* = 135)	
Yes	67 (50%)

*Note. SD, Standard Deviation; ED, Eating Disorder; OSFED, Other Specified Feeding and Eating Disorder; ARFID, Avoidant/Restrictive Food Intake Disorder; BMI, Body Mass Index; HLOC, Higher Level of ED Care*

### Multivariate results (see Table 2)

1. *Association of Malnutrition with Anxiety and Depression Symptoms*

**Table 2:** Associations of malnutrition, eating disorder duration, and pre-morbid weight status with anxiety and depression symptoms

	Depression			Anxiety		
Model	β	95% CI	*p*-value	β	95% CI	p-value
Malnutrition^a^						
Weight restored (*ref*)	Ref	Ref	Ref	Ref	Ref	Ref
Mild/moderate malnutrition	0.48	−5.22, 6.17	0.87	0.07	−2.39, 2.53	0.96
Moderate/severe malnutrition	−0.10	−6.48, 6.28	0.98	0.02	−2.74, 2.78	0.99
ED duration on^b^						
≤ 6 months (*ref*)	Ref	Ref	Ref	Ref	Ref	Ref
7–12 months	8.84	0.52, 17.15	0.038*	1.62	−1.98, 5.21	0.38
> 1 year	6.33	−0.23, 12.89	0.059	2.59	−0.23, 5.41	0.071
Pre-morbid weight status^c^						
< 75th%ile BMI (*ref*)	Ref	Ref	Ref	Ref	Ref	Ref
≥ 75th%ile BMI	−7.18	−12.87, −1.48	0.014*	−2.27	−4.70, 0.16	0.067

^a^Adjusted for age, sex assigned at birth, sexual orientation, ED diagnosis, and use of psychiatric medication

^b^Adjusted for malnutrition, HLOC, and time under our care

^c^Adjusted for age, malnutrition, time under our care, and ED diagnosis

*Statistically significant at *p* < 0.05

*Note. CI, Confidence Interval; ED, eating disorder; BMI, body mass index*

Degree of malnutrition was not associated with either anxiety [mild/moderate vs. weight restored (β = 0.07; 95% CI [− 2.39, 2.53]; *p* = 0.96); moderate/severe vs. weight restored (β = 0.02; 95% CI [− 2.74, 2.78]; *p* = 0.99)] or depression symptoms [mild/moderate vs. weight restored (β = 0.48; 95% CI [− 5.22, 6.17]; *p* = 0.87); moderate/severe vs. weight restored (β = − 0.10; 95% CI [− 6.48, 6.28]; *p* = 0.98)] after adjusting for age, sex assigned at birth, sexual orientation, ED diagnosis (AN, OSFED or ARFID), and use of psychiatric medication.

2. *Association of Duration of Symptoms with Anxiety and Depression Symptoms*

**Fig. 1:**
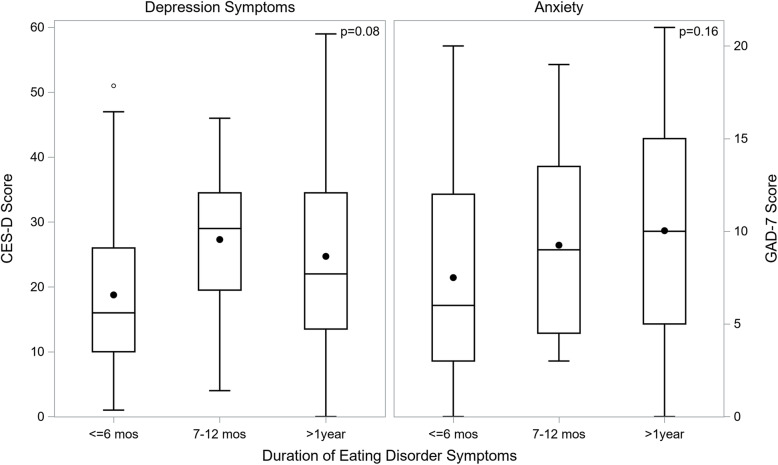
The Unadjusted Differences in Anxiety and Depression Symptoms by Duration of Eating Disorder Symptoms

In bivariate analyses, duration of symptoms was not associated with anxiety (*p* = 0.16) or depression (*p* = 0.08), though a trend is notable of increased anxiety scores as the duration of ED symptoms increases (Fig. 1). In our multivariate regressions analyses, compared to those with the shortest duration of ED symptoms (≤6 months), those with longer duration of symptoms had higher GAD-7 scores [7–12 months (β = 1.62; 95% CI [− 1.98, 5.21]; *p =* 0.375) and > 1 year (β = 2.59; 95% CI [− 0.23, 5.41]; *p* = 0.071)] after adjusting for malnutrition, HLOC, and time under our care, though the results were not statistically different. Compared to those with the shortest length of ED symptoms (≤6 months), patients with symptoms for 7–12 months had significantly higher CES-D scores (β = 8.84; 95% CI [0.52, 17.15], *p* = 0.038) after adjusting for malnutrition, HLOC, and time under our care. Patients with symptoms > 1 year also had higher CESD scores than those with symptoms ≤6 months, though the difference was not significant (β = 6.33; 95% CI [− 0.23, 12.89]; *p* = 0.059).

3. *Associations of Pre-morbid BMI status with Anxiety and Depression Symptoms*

**Fig. 2:**
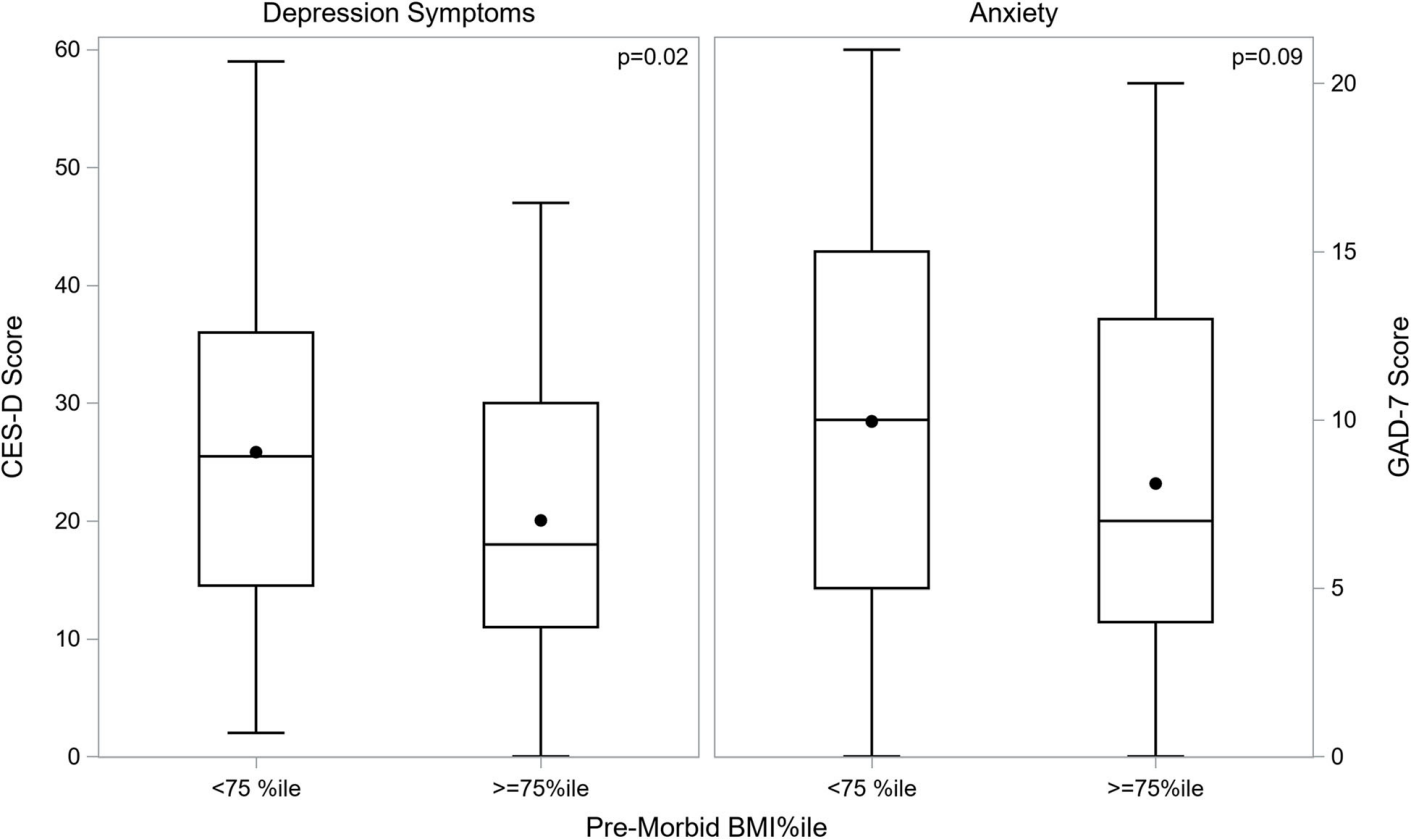
The Unadjusted Differences in Anxiety and Depression Symptoms by Pre-Morbid BMI percentile

In bivariate analyses, pre-morbid BMI percentile was significantly associated with depression symptoms (*p* = 0.02) but not anxiety (*p* = 0.09), though a similar trend is noted for both measures (Fig. 2). After adjusting for age, malnutrition, time under our care, and ED diagnosis, those with premorbid BMIs ≥75th %ile had significantly lower CES-D scores (β = − 7.18; 95% CI [− 12.87, − 1.48]; *p* = 0.014) than those who had premorbid BMIs <75th %ile. They also had lower GAD-7 scores, though the difference was not statistically significant (β = − 2.27; 95% CI [− 4.70, 0.16]; *p* = 0.067).

## Discussion

Our study is one of the first to explore the association of multiple restrictive ED characteristics (malnutrition, duration of ED symptoms, and pre-morbid BMI status) with co-morbid anxiety and depression symptoms. The majority of the study participants met or exceeded the cutoffs for clinical anxiety and depression, demonstrating a high rate of clinically significant anxiety and depression in adolescents and young adults with restrictive EDs. We did not find that degree of malnutrition was associated with symptoms of anxiety and depression, after controlling for age, sex assigned at birth, sexual orientation, ED diagnosis, and use of psychiatric medication. Though this was not as we hypothesized, this highlights the importance of other factors that contribute to depression and anxiety in this vulnerable population. Longer duration of ED illness showed a trend towards clinically worse depression and anxiety symptoms, though the results were not statistically significant. However, this finding demonstrates a need for rapid identification and treatment of EDs and its co-morbid mental health disorders. Lastly, contrary to our hypothesis, those who had a pre-morbid BMI ≥75th percentile had lower depression scores than those who had BMIs <75th percentile prior to weight loss. Though the association with anxiety symptoms was not statistically significant, the trend noted is similar and still clinically relevant, and an important factor to study further.

It is critical for us to determine whether weight restoration should remain the top priority in ED treatment, or whether optimal treatment includes balanced focus on physical and psychological components of EDs. Some studies of patients with AN have found an association between severity of malnutrition and anxiety/depressive symptoms [19], and that weight restoration led to improved mental health [19–23]. However, like us, others have found no statistically significant relationship between degree of malnutrition and depressive [24, 25] or anxiety symptoms [25], and found that these symptoms may persist despite weight restoration [19, 39, 40]. Given the dangers associated with struggling with both an ED and comorbid psychiatric illness [14, 16, 17], these inconsistent findings highlight the need for more comprehensive ED treatment that focuses on *both* nutritional rehabilitation and mental health treatment.

Our study found that longer chronicity of ED symptoms may trend towards more severe depression and anxiety symptoms. This finding is important to consider in treatment for EDs as negative affect and internalizing symptoms can contribute to more severe ED symptoms [15], and depression is also associated with lower rates of ED recovery over time [41, 42]. Longer duration of ED illness is overall associated with worse prognosis and treatment outcomes (e.g., weight gain and ED recovery) among individuals diagnosed with AN [13, 15, 43–45]. However, research on the effect of duration of illness on co-morbid anxiety and depression for individuals with restrictive EDs is generally limited. Our finding is in line with a previous study which showed that depression and anxiety scores were significantly higher for females with AN who had a longer duration of illness [45]. And findings on duration of ED illness consistently suggest that longer duration raises the risk for worse psychological symptoms, worse prognosis, treatment outcomes, and even mortality [46]. Our study highlights the need for early identification and intervention for individuals with restrictive EDs due to the possibility of worsening depression and anxiety with a longer duration of illness. Our findings also suggest that adapting therapeutic approaches based on the illness duration may be necessary given the association between the duration of illness and psychological correlates of restrictive EDs.

Lastly, our cohort of adolescents and young adults with higher pre-morbid BMIs actually had *lower* depression scores at the time of their baseline surveys. Anxiety scores showed a similar trend but was not significant. Of note, we used a cut off of 75th percentile due to sample size and clinical suspicion that those with a BMI 75th to 85th percentile had similar weight-stigma experiences compared to those who met CDC criteria for overweight/obese (≥85th percentile). A sensitivity analysis using the cutoff of 85th percentile had similar findings as the cutoff of 75th percentile (though it was not statistically significant, possibly due to sample size). Potentially, this finding is actually a reflection of improved anxiety and depression after weight loss, as those with higher weights tend to experience more weight stigma and elevated mental health concerns prior to weight loss [47, 48]. Youth with overweight or obesity often experience weight stigma as weight-based victimization, teasing, and bullying [49], and those with restrictive EDs with a higher pre-morbid weight status may experience social reinforcement of their disordered eating behaviors; often patients receive praise as they begin their weight loss [50]. Other studies have shown that patients with severe obesity experience improved anxiety and depression with a psychosocial weight loss intervention [51] or bariatric surgery [52, 53]. However, we were not able to find studies that have observed the effects of malnutrition, ED treatment, or weight gain on the levels of anxiety and depression in this unique population of patients with higher pre-morbid weights and a restrictive ED. The temporal relationship of the mental health comorbidities in these patients could be enlightening and provide insight on effective ED treatment approaches and priorities for this population.

Our study was unique in that our cohort included participants with not just AN, but also other EDs affected by malnutrition, such as OSFED and ARFID. Additionally, our use of eBMI, based on each patient’s previous growth trajectory, allowed us to capture the effects of *relative* malnutrition. This is vital for capturing the spectrum of patients who are at different weights. However, our study was not without limitations. The sample size was not large enough to include all variables that could be of clinical significance, so for our third model, a stepwise approach was used to include the most statistically influential variables. There was also large variability in degree of malnutrition, length of time under our care when recruited, and previous experiences with intensive ED treatment. This may have a role in why a cross-sectional association between malnutrition and anxiety and depression symptoms was not found. Additionally, as a cross-sectional study, we were unable to capture individuals’ changes in anxiety and depression symptoms in regards to changes in weight during treatment, a critical question in need of clarification. Therefore, a longitudinal study could be vital for answering many questions. It could demonstrate how weight restoration and rapidity of treatment aid in the treatment of anxiety and depression symptoms in patients with EDs. Additionally, a longitudinal study would allow us to explore how anxiety and depressive symptoms change throughout treatment for all patients, and especially those with high pre-morbid weight statuses.

## Conclusions

Our study showed that adolescents and young adults with restrictive EDs are at high risk for anxiety and depression and that severity of malnutrition may not be an independent predictor of the level of anxiety and depression. Many factors are critical for improving treatment outcomes and the chance of ED recovery. We identified that duration of ED symptoms and pre-morbid weight status are two potentially important factors. Other factors that may contribute to severity of psychological symptoms include pre-existing or co-occurring depression or anxiety [54, 55], or simply the severity of the ED itself—as more severe ED pathology may be associated with more significant depression and anxiety [55, 56]. Additionally, the literature provides evidence that genetic factors may increase patients’ risk of disordered eating, depression, and even suicide [57], and hormonal factors such as cortisol and leptin may also have a role in restrictive eating and mental healt h[58, 59]. Restoring weight and addressing malnutrition may not be the sole factors for full ED recovery. Above all, it is critical that we identify restrictive EDs and mental health co-morbidities in patients of any weight and that we quickly and efficiently get our patients into comprehensive, multidisciplinary ED treatment.

## Data Availability

The datasets generated and/or analyzed during the current study are not publicly available due to protection of participants’ privacy, but may be available from the corresponding author on reasonable request with IRB approval.

## Abbreviations

AAN: Atypical Anorexia Nervosa
AN: Anorexia Nervosa
ARFID: Avoidant/restrictive Food Intake Disorder
BCH: Boston Children’s Hospital
BMI: Body Mass Index
CDC: Centers for Disease Control and Prevention
CES-D: Center for Epidemiologic Studies Depression Scale
CI: Confidence Interval
ED: Eating Disorder
FBT: Family Based Treatment
GAD-7: Generalized Anxiety Disorder 7-item Scale
HLOC: Higher Level of Eating Disorder Care
OSFED: Other Specified Feeding and Eating Disorder
RECOVERY: Registry of Eating Disorders and their Co-morbidities OVER time in Youth
SD: Standard Deviation
